# (–)-(*S*)-*N*,*N*′-Bis[1-(1-naphth­yl)eth­yl]­oxalamide

**DOI:** 10.1107/S1600536810043424

**Published:** 2010-10-31

**Authors:** Sylvain Bernès, Guadalupe Hernández, Jaime Vázquez, Alejandra Tovar, René Gutiérrez

**Affiliations:** a DEP Facultad de Ciencias Químicas, UANL, Guerrero y Progreso S/N, Col. Treviño, 64570 Monterrey, NL, Mexico; b Laboratorio de Síntesis de Complejos, Facultad de Ciencias Químicas, Universidad Autónoma de Puebla, AP 1067, 72001 Puebla, Pue., Mexico; c Universidad de la Cañada, Cd. Universitaria, 68540, Teotitlán de Flores Magón, Oax., Mexico

## Abstract

The title mol­ecule, C_26_H_24_N_2_O_2_, displays *C*_2_ symmetry, with the mol­ecule located on a twofold axis perpendicular to the plane of the oxalamide unit –NH—CO—CO—NH–. The oxalamide core deviates from planarity, as reflected by the O=C—C=O and N—C—C—N torsion angles of 164.3 (5) and 163.2 (5)°, respectively. The naphthyl groups are oriented toward the same face of the oxalamide mean plane and make a dihedral angle of 43.76 (8)°. This conformation is suitable for the formation of inter­molecular N—H⋯O hydrogen bonds, giving noncentrosymmetric dimers incorporating *R*_2_^2^(10) ring motifs. These nonbonding inter­actions propagate along the 6_1_ screw axis normal to the mol­ecular twofold axis, resulting in a single-stranded right-handed helix parallel to [001]. In the crystal, Δ helices are arranged side-by-side and inter­act through π–π contacts between naphthyl groups. The shortest centroid–centroid separation between inter­acting benzene rings is 3.623 (4) Å.

## Related literature

For crystal structures of closely related oxalamides, see: Štefanić *et al.* (2003 ▶); Zhang *et al.* (2006 ▶); Lee & Wang (2007 ▶); Lee (2010 ▶). For general references on dicarboxamides and oxalamides, and their synthesis under solvent-free conditions, see: Bernès *et al.* (2008 ▶); Montero-Vázquez *et al.* (2008 ▶); Jeon *et al.* (2005 ▶); Noyori (2005 ▶). For helicity assignment in enanti­omorphic space groups, see: Ha & Allewell (1997 ▶).
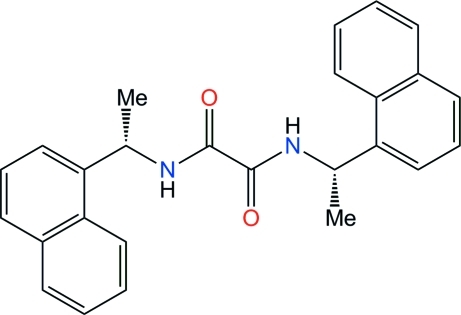


## Experimental

### 

#### Crystal data

C_26_H_24_N_2_O_2_
*M* *_r_* = 396.47
Hexagonal, 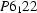

*a* = 11.4489 (11) Å
*c* = 28.350 (4) Å
*V* = 3218.2 (7) Å^3^
*Z* = 6
Mo *K*α radiation
μ = 0.08 mm^−1^
*T* = 298 K
0.40 × 0.22 × 0.20 mm

#### Data collection

Siemens *P*4 diffractometer
5314 measured reflections
1208 independent reflections
730 reflections with *I* > 2σ(*I*)
*R*_int_ = 0.057
3 standard reflections every 97 reflections  intensity decay: 1%

#### Refinement

*R*[*F* ^2^ > 2σ(*F* ^2^)] = 0.043
*wR*(*F* ^2^) = 0.138
*S* = 1.11
1208 reflections
141 parameters
1 restraint
H atoms treated by a mixture of independent and constrained refinement
Δρ_max_ = 0.12 e Å^−3^
Δρ_min_ = −0.14 e Å^−3^

### 

Data collection: *XSCANS* (Siemens, 1996 ▶); cell refinement: *XSCANS*; data reduction: *XSCANS*; program(s) used to solve structure: *SHELXS97* (Sheldrick, 2008 ▶); program(s) used to refine structure: *SHELXL97* (Sheldrick, 2008 ▶); molecular graphics: *Mercury* (Macrae *et al.*, 2006 ▶); software used to prepare material for publication: *SHELXL97*.

## Supplementary Material

Crystal structure: contains datablocks I, global. DOI: 10.1107/S1600536810043424/jh2225sup1.cif

Structure factors: contains datablocks I. DOI: 10.1107/S1600536810043424/jh2225Isup2.hkl

Additional supplementary materials:  crystallographic information; 3D view; checkCIF report

## Figures and Tables

**Table 1:** Hydrogen-bond geometry (Å, °)

*D*—H⋯*A*	*D*—H	H⋯*A*	*D*⋯*A*	*D*—H⋯*A*
N2—H2⋯O1^i^	0.91 (1)	2.06 (2)	2.931 (3)	161 (3)

Symmetry code: (i) 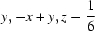.

**Fig. 1.**
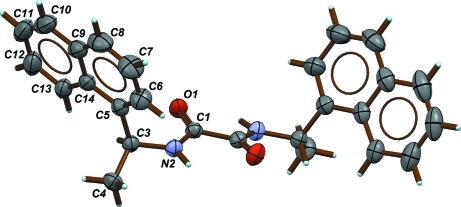
The title molecule with displacement ellipsoids for non-H atoms shown at the 30% probability level. Non labeled atoms are generated by symmetry operator x-y, -y, 1 - z.

**Fig. 2.**
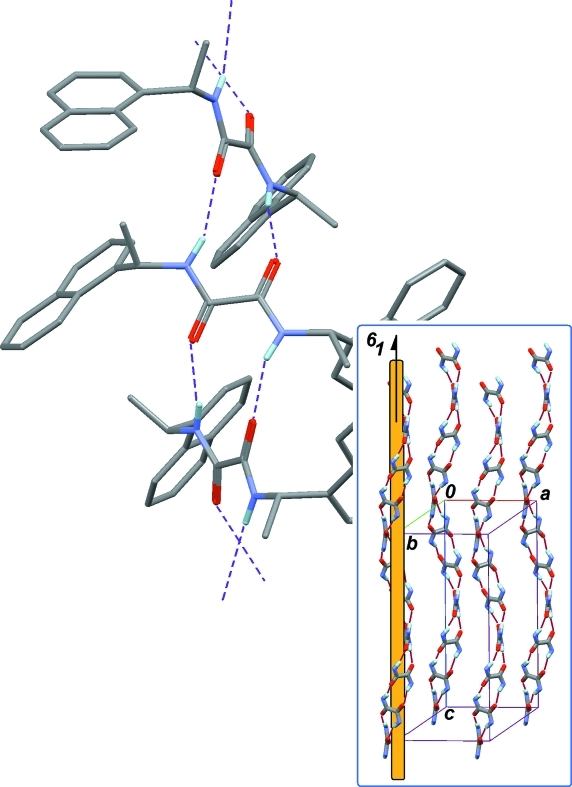
Hydrogen bonds linking molecules in the [001] direction (dashed bonds). The inset represents Δ helix formed by hydrogen bonds along the screw axis 61. For the sake of clarity, only oxalamide atoms are represented, and one 61 axis is shown (yellow backbone).

## supplementary crystallographic information

### Comment

Earlier, we have focused our attention on dicarboxamides (Bernès *et al.*, 2008), mostly due to their versatile and interesting properties. In continuation of this work, we synthesized the title chiral compound, under the solvent-free approach, because the reactions occur under mild conditions and usually require easier workup procedures and simpler equipment. Likewise, reducing the use of organic solvents and minimizing the formation of waste are worth-considering points to keep in mind by using this protocol (Jeon *et al.*, 2005; Noyori, 2005).

The title molecule belongs to the oxalamide family, a well studied class of compounds, which are useful in many areas (*e.g*. Montero-Vázquez *et al.*, 2008). The molecule is placed on a twofold axis, passing by the midpoint of the central C1—C1*^i^* bond (symmetry code *i*: *x*-*y*, -*y*, 1 - *z*). The twofold axis is perpendicular to the mean plane of the core oxalamide group. In contrast to other oxalamide derivatives (*e.g*. Zhang *et al.*, 2006) the oxalamide group in the title compound is not planar. As a consequence, *trans* angles O1—C1—C1*^i^*—O1*^i^* and N2—C1—C1*^i^*—N2*^i^* deviate significantly from 180°. N atoms are substituted by chiral groups including naphthyl cycles, which make a dihedral angle of 43.76 (8)° (Fig. 1).

The *C*_2_-symmetric molecules are connected in the crystal through N2—H2···O1*^ii^* hydrogen bonds (symmetry code *ii*: *y*, -*x* + *y*, *z* - 1/6), which generate *R*_2_^2^(10) ring motifs (Fig. 2). Such motifs have been observed in related chiral (Štefanić *et al.*, 2003) and achiral (Lee & Wang, 2007) oxalamides, but gave different supramolecular structures, depending of the ability of terminal groups to be involved in hydrogen bonds. In the case of the title oxalamide, hydrogen bonds form a supramolecular structure based on a single stranded helix using the screw axis 6_1_ as backbone (Fig. 2, inset). The molecular and supramolecular axes are thus perpendicular. Although the space group is enantiomorphic, the helicity of the supramolecular structure can be assigned, since the chirality of the asymmetric unit is known (Ha & Allewell, 1997). The *S* configuration of chiral center C3 affords right-handed helix oriented along [001]. It is worth noting that such chiral supramolecular structures cannot be achieved for centrosymmetric oxalamide derivatives (Zhang *et al.*, 2006; Lee, 2010).

The crystal structure of the title compound is build up on densely packed parallel Δ helix, which interact through π–π contacts involving terminal naphthyl groups. The shortest intermolecular separation between symmetry-related benzene rings is 3.623 (4) Å [*Cg*···*Cg^iii^*, where *Cg* is the centroid of ring C9···C14 and *iii* is symmetry code: 1 - *y*, 1 - *x*, 5/6 - *z*]. This contact should however be regarded as a secondary interaction compared to hydrogen bonds forming the one-dimensional supramolecular structure. Although the ring approach is short, constraints induced by molecular conformation reduce the efficiency of such contacts. For instance, π systems involved in π–π contacts are not parallel, making a dihedral angle of 11.7 (2)°.

### Experimental

Under solvent-free conditions, a mixture of oxalyl chloride (0.2 g, 1.5 mmol) and (*S*)-(–)-1-(1-naphthyl)ethylamine (0.53 g, 3.0 mmol) in a 1:2 molar ratio were mixed at room temperature, giving a white solid. The crude was recrystallized twice from CH_2_Cl_2_, affording colorless crystals of the title compound. Yield 96%; m.p. 240–242 °C. Spectroscopic data: [α]^25^_D_ = -10.5 (*c* 1, CHCl_3_). FT—IR (KBr): 3450 cm^-1^ (NH), 1650 cm^-1^ (CO). ^1^H NMR (400 MHz, CDCl_3_/TMS): δ = 1.5–1.7 (d, 6H, CHCH_3_), 4.1 (br s, 2H, NH), 5.1 (q, 2H, CHCH_3_), 7.3–8.1 (m, 14 H, Ar); ^13^C NMR (100 MHz, CDCl_3_/TMS) δ = 23.3 (CCH_3_), 46.9 (CHCH_3_), 122.7 (Ar), 125.1 (Ar), 125.6 (Ar), 127.9 (Ar), 128.9 (Ar), 128.4 (Ar), 133.7 (Ar), 138.9.(Ar), 144.3 (Ar), 158.5 (C=O). MS—EI: *m*/*z* = 396 (*M*^+^).

### Refinement

All C-bonded H atoms were placed in idealized positions and refined as riding to their carrier C atoms, with bond lengths fixed to 0.93 (aromatic CH), 0.96 (methyl CH_3_), and 0.98 Å (methine CH). Atom H2 bonded to N2 was found in a difference map and refined freely, although N—H bond length was restrained to 0.91 (1) Å. Isotropic displacement parameters were calculated as *U*_iso_(H) = 1.5*U*_eq_(carrier atom) for the methyl group and *U*_iso_(H) = 1.2*U*_eq_(carrier atom) otherwise. The absolute configuration was assigned from the known configuration of the chiral amine used as starting material, and measured Friedel pairs (705) were merged.

### Crystal data

**Table:**

C_26_H_24_N_2_O_2_	*D*_x_ = 1.227 Mg m^−^^3^
*M**_r_* = 396.47	Melting point: 513 K
Hexagonal, *P*6_1_22	Mo *K*α radiation, λ = 0.71073 Å
Hall symbol: P 61 2 (0 0 -1)	Cell parameters from 67 reflections
*a* = 11.4489 (11) Å	θ = 4.1–11.9°
*c* = 28.350 (4) Å	µ = 0.08 mm^−^^1^
*V* = 3218.2 (7) Å^3^	*T* = 298 K
*Z* = 6	Prism, colourless
*F*(000) = 1260	0.40 × 0.22 × 0.20 mm

### Data collection

**Table:**

Siemens P4 diffractometer	*R*_int_ = 0.057
Radiation source: fine-focus sealed tube	θ_max_ = 25.1°, θ_min_ = 2.1°
graphite	*h* = −13→2
ω scans	*k* = −1→13
5314 measured reflections	*l* = −33→1
1208 independent reflections	3 standard reflections every 97 reflections
730 reflections with *I* > 2σ(*I*)	intensity decay: 1%

### Refinement

**Table:**

Refinement on *F*^2^	Secondary atom site location: difference Fourier map
Least-squares matrix: full	Hydrogen site location: inferred from neighbouring sites
*R*[*F*^2^ > 2σ(*F*^2^)] = 0.043	H atoms treated by a mixture of independent and constrained refinement
*wR*(*F*^2^) = 0.138	*w* = 1/[σ^2^(*F*_o_^2^) + (0.0649*P*)^2^ + 0.2213*P*] where *P* = (*F*_o_^2^ + 2*F*_c_^2^)/3
*S* = 1.11	(Δ/σ)_max_ < 0.001
1208 reflections	Δρ_max_ = 0.12 e Å^−^^3^
141 parameters	Δρ_min_ = −0.14 e Å^−^^3^
1 restraint	Extinction correction: *SHELXL97* (Sheldrick, 2008), Fc^*^=kFc[1+0.001xFc^2^λ^3^/sin(2θ)]^-1/4^
0 constraints	Extinction coefficient: 0.011 (2)
Primary atom site location: structure-invariant direct methods	

### Fractional atomic coordinates and isotropic or equivalent isotropic displacement parameters (Å^2^)

**Table:**

	*x*	*y*	*z*	*U*_iso_*/*U*_eq_	
O1	0.1760 (3)	0.1496 (2)	0.53227 (7)	0.0718 (8)	
C1	0.1517 (3)	0.0760 (3)	0.49767 (11)	0.0539 (8)	
N2	0.1898 (3)	0.1213 (3)	0.45411 (9)	0.0594 (8)	
H2	0.168 (3)	0.058 (3)	0.4311 (9)	0.071*	
C3	0.2678 (3)	0.2653 (3)	0.44315 (11)	0.0589 (9)	
H3A	0.3249	0.3110	0.4705	0.071*	
C4	0.3604 (4)	0.2880 (4)	0.40144 (13)	0.0852 (13)	
H4B	0.4194	0.2535	0.4088	0.128*	
H4C	0.4131	0.3828	0.3949	0.128*	
H4D	0.3072	0.2420	0.3743	0.128*	
C5	0.1787 (4)	0.3254 (4)	0.43491 (11)	0.0632 (10)	
C6	0.0662 (4)	0.2582 (5)	0.40749 (15)	0.0878 (12)	
H6A	0.0436	0.1736	0.3957	0.105*	
C7	−0.0158 (6)	0.3123 (7)	0.39661 (18)	0.1117 (17)	
H7A	−0.0907	0.2651	0.3773	0.134*	
C8	0.0142 (6)	0.4343 (7)	0.4144 (2)	0.1100 (17)	
H8B	−0.0420	0.4692	0.4079	0.132*	
C9	0.1289 (6)	0.5082 (5)	0.44246 (16)	0.0855 (13)	
C10	0.1624 (8)	0.6363 (6)	0.4601 (2)	0.119 (2)	
H10B	0.1074	0.6721	0.4529	0.143*	
C11	0.2721 (8)	0.7080 (6)	0.4873 (2)	0.126 (2)	
H11D	0.2911	0.7913	0.4993	0.151*	
C12	0.3575 (6)	0.6564 (5)	0.49739 (19)	0.1110 (17)	
H12B	0.4343	0.7068	0.5156	0.133*	
C13	0.3293 (5)	0.5332 (4)	0.48084 (13)	0.0781 (12)	
H13B	0.3868	0.5003	0.4881	0.094*	
C14	0.2145 (4)	0.4552 (4)	0.45291 (12)	0.0660 (10)	

### Atomic displacement parameters (Å^2^)

**Table:**

	*U*^11^	*U*^22^	*U*^33^	*U*^12^	*U*^13^	*U*^23^
O1	0.0854 (19)	0.0634 (16)	0.0510 (13)	0.0256 (14)	0.0089 (12)	−0.0117 (12)
C1	0.055 (2)	0.0567 (19)	0.0495 (17)	0.0279 (17)	0.0013 (16)	−0.0044 (19)
N2	0.071 (2)	0.0555 (19)	0.0477 (15)	0.0284 (16)	0.0035 (15)	−0.0042 (13)
C3	0.063 (2)	0.056 (2)	0.0540 (18)	0.0272 (19)	0.0031 (17)	−0.0023 (16)
C4	0.088 (3)	0.084 (3)	0.081 (2)	0.041 (3)	0.032 (2)	0.011 (2)
C5	0.068 (3)	0.068 (3)	0.0532 (18)	0.033 (2)	0.0073 (19)	0.0067 (19)
C6	0.087 (3)	0.093 (3)	0.087 (3)	0.048 (3)	−0.015 (3)	−0.001 (3)
C7	0.097 (4)	0.136 (5)	0.113 (4)	0.067 (4)	−0.017 (3)	0.014 (4)
C8	0.108 (4)	0.136 (5)	0.120 (4)	0.087 (4)	0.018 (4)	0.035 (4)
C9	0.104 (4)	0.087 (3)	0.081 (3)	0.060 (3)	0.034 (3)	0.026 (3)
C10	0.175 (7)	0.104 (4)	0.116 (4)	0.098 (5)	0.066 (4)	0.037 (4)
C11	0.178 (7)	0.073 (4)	0.128 (5)	0.065 (4)	0.061 (5)	0.013 (3)
C12	0.129 (5)	0.065 (3)	0.110 (3)	0.027 (3)	0.030 (4)	−0.003 (3)
C13	0.089 (3)	0.058 (3)	0.075 (2)	0.028 (2)	0.015 (2)	−0.002 (2)
C14	0.078 (3)	0.063 (2)	0.0587 (19)	0.036 (2)	0.025 (2)	0.014 (2)

### Geometric parameters (Å, °)

**Table:**

O1—C1	1.231 (4)	C7—C8	1.358 (7)
C1—N2	1.326 (4)	C7—H7A	0.9300
C1—C1^i^	1.512 (7)	C8—C9	1.400 (7)
N2—C3	1.463 (4)	C8—H8B	0.9300
N2—H2	0.911 (10)	C9—C10	1.409 (7)
C3—C5	1.506 (5)	C9—C14	1.418 (6)
C3—C4	1.522 (5)	C10—C11	1.348 (8)
C3—H3A	0.9800	C10—H10B	0.9300
C4—H4B	0.9600	C11—C12	1.401 (7)
C4—H4C	0.9600	C11—H11D	0.9300
C4—H4D	0.9600	C12—C13	1.363 (6)
C5—C6	1.366 (5)	C12—H12B	0.9300
C5—C14	1.424 (5)	C13—C14	1.406 (6)
C6—C7	1.394 (6)	C13—H13B	0.9300
C6—H6A	0.9300		
			
O1—C1—N2	123.8 (3)	C8—C7—C6	119.6 (5)
O1—C1—C1^i^	121.4 (4)	C8—C7—H7A	120.2
N2—C1—C1^i^	114.8 (3)	C6—C7—H7A	120.2
C1—N2—C3	122.3 (3)	C7—C8—C9	120.8 (5)
C1—N2—H2	117 (2)	C7—C8—H8B	119.6
C3—N2—H2	121 (2)	C9—C8—H8B	119.6
N2—C3—C5	112.1 (3)	C8—C9—C10	121.0 (6)
N2—C3—C4	109.8 (3)	C8—C9—C14	120.0 (4)
C5—C3—C4	112.0 (3)	C10—C9—C14	119.0 (5)
N2—C3—H3A	107.6	C11—C10—C9	121.5 (6)
C5—C3—H3A	107.6	C11—C10—H10B	119.2
C4—C3—H3A	107.6	C9—C10—H10B	119.2
C3—C4—H4B	109.5	C10—C11—C12	119.6 (6)
C3—C4—H4C	109.5	C10—C11—H11D	120.2
H4B—C4—H4C	109.5	C12—C11—H11D	120.2
C3—C4—H4D	109.5	C13—C12—C11	120.8 (6)
H4B—C4—H4D	109.5	C13—C12—H12B	119.6
H4C—C4—H4D	109.5	C11—C12—H12B	119.6
C6—C5—C14	119.4 (4)	C12—C13—C14	120.9 (5)
C6—C5—C3	119.6 (3)	C12—C13—H13B	119.6
C14—C5—C3	120.9 (3)	C14—C13—H13B	119.6
C5—C6—C7	122.0 (5)	C13—C14—C9	118.2 (4)
C5—C6—H6A	119.0	C13—C14—C5	123.7 (4)
C7—C6—H6A	119.0	C9—C14—C5	118.1 (4)
			
O1—C1—N2—C3	1.3 (5)	C8—C9—C10—C11	179.7 (5)
O1—C1—C1^i^—O1^i^	164.3 (5)	C14—C9—C10—C11	−1.2 (7)
C1^i^—C1—N2—C3	−178.1 (2)	C9—C10—C11—C12	1.7 (8)
C1—N2—C3—C5	−86.9 (4)	C10—C11—C12—C13	−1.4 (8)
C1—N2—C3—C4	148.0 (3)	C11—C12—C13—C14	0.5 (7)
N2—C3—C5—C6	−44.7 (4)	C12—C13—C14—C9	0.0 (5)
N2—C1—C1^i^—N2^i^	163.2 (5)	C12—C13—C14—C5	−179.4 (4)
C4—C3—C5—C6	79.2 (4)	C8—C9—C14—C13	179.4 (4)
N2—C3—C5—C14	139.2 (3)	C10—C9—C14—C13	0.3 (5)
C4—C3—C5—C14	−96.9 (4)	C8—C9—C14—C5	−1.2 (5)
C14—C5—C6—C7	−0.3 (6)	C10—C9—C14—C5	179.8 (4)
C3—C5—C6—C7	−176.5 (4)	C6—C5—C14—C13	−179.0 (3)
C5—C6—C7—C8	−1.5 (8)	C3—C5—C14—C13	−2.9 (5)
C6—C7—C8—C9	1.9 (8)	C6—C5—C14—C9	1.6 (5)
C7—C8—C9—C10	178.5 (5)	C3—C5—C14—C9	177.7 (3)
C7—C8—C9—C14	−0.6 (7)		

Symmetry codes: (i) *x*−*y*, −*y*, −*z*+1.

### Hydrogen-bond geometry (Å, °)

**Table:**

*D*—H···*A*	*D*—H	H···*A*	*D*···*A*	*D*—H···*A*
N2—H2···O1^ii^	0.91 (1)	2.06 (2)	2.931 (3)	161 (3)

Symmetry codes: (ii) *y*, −*x*+*y*, *z*−1/6.
